# Cancer chemotherapy: insights into cellular and tumor microenvironmental mechanisms of action

**DOI:** 10.3389/fonc.2022.960317

**Published:** 2022-07-29

**Authors:** Caitlin M. Tilsed, Scott A. Fisher, Anna K. Nowak, Richard A. Lake, W. Joost Lesterhuis

**Affiliations:** ^1^ National Centre for Asbestos Related Diseases, Institute for Respiratory Health, Nedlands, WA, Australia; ^2^ School of Biomedical Sciences, University of Western Australia, Crawley, WA, Australia; ^3^ Medical School, University of Western Australia, Crawley, WA, Australia; ^4^ Department of Medical Oncology, Sir Charles Gairdner Hospital, Nedlands, WA, Australia; ^5^ Telethon Kids Institute, University of Western Australia, West Perth, WA, Australia

**Keywords:** chemotherapy, tumor microenvironment, cell death, immune response, mechanism of action, cancer chemotherapy

## Abstract

Chemotherapy has historically been the mainstay of cancer treatment, but our understanding of what drives a successful therapeutic response remains limited. The diverse response of cancer patients to chemotherapy has been attributed principally to differences in the proliferation rate of the tumor cells, but there is actually very little experimental data supporting this hypothesis. Instead, other mechanisms at the cellular level and the composition of the tumor microenvironment appear to drive chemotherapy sensitivity. In particular, the immune system is a critical determinant of chemotherapy response with the depletion or knock-out of key immune cell populations or immunological mediators completely abrogating the benefits of chemotherapy in pre-clinical models. In this perspective, we review the literature regarding the known mechanisms of action of cytotoxic chemotherapy agents and the determinants of response to chemotherapy from the level of individual cells to the composition of the tumor microenvironment. We then summarize current work toward the development of dynamic biomarkers for response and propose a model for a chemotherapy sensitive tumor microenvironment.

## Introduction

The discovery and development of cytotoxic chemotherapies undoubtedly changed the landscape of cancer treatment. The first indication that a chemotherapy, or a cytotoxic chemical, could be a potential treatment for cancer was an incidental observation made in individuals exposed to a biochemical weapon, mustard gas. Soldiers exposed to mustard gas experienced severe leukopenia and marked depletion of the bone marrow and lymph nodes (1). This discovery was translated to one of the commonly studied cancers of that period, lymphoma, with the treatment resulting in significant, albeit temporary, regression. This stimulated the development and implementation of drug screening programs that tested multiple compounds *in vitro* for anti-cancer properties (2). Promising compounds were moved into animal cancer models, clinical trials in patients and developed into our current oncological treatment paradigm.

**Figure 1:**
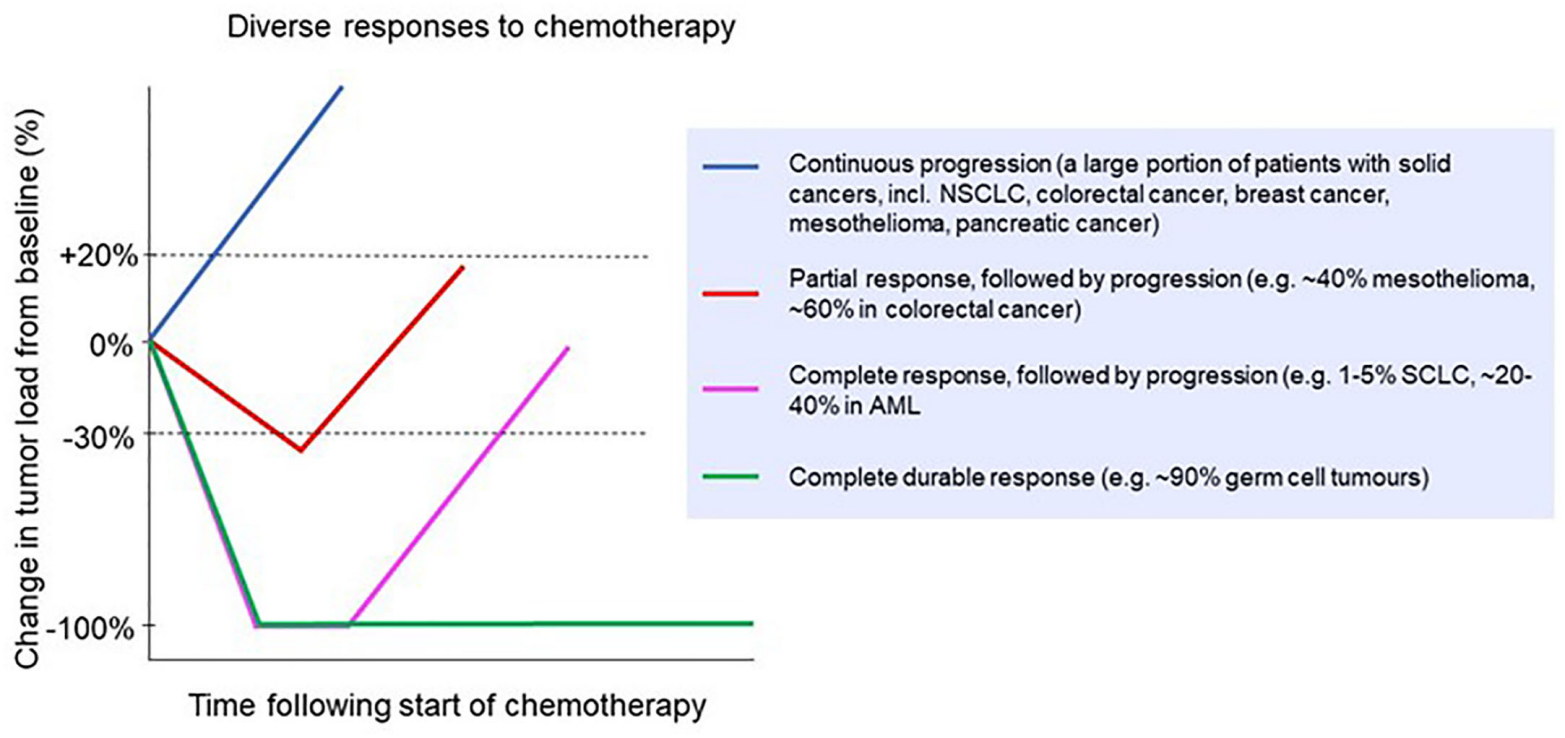
Clinical responses to chemotherapy in a range of cancer types. Patients might experience no response (continuous progression) or a partial response followed by progression [e.g. non-small cell lung cancer (9), colorectal cancer (10) breast cancer (11), mesothelioma (12) and pancreatic cancer (13)]; a complete response followed by progression [e.g. small cell lung cancer (14) and acute myeloid leukemia (15)]; or a complete durable response [e.g. germ cell tumors (16)].

Despite the development of new treatment modalities, including oncogene-targeted therapies such as tyrosine kinase inhibitors, and immunotherapies such as immune checkpoint inhibitors, chemotherapy remains the first-line treatment for many cancers. In fact, despite the global search for new therapies that work synergistically with immune checkpoint blockade, combinations with classic chemotherapy so far have shown the best results (3). In many localized cancers, chemotherapy before or after surgery and/or combined with radiotherapy can provide durable, long-term survival benefits for many patients, such as chemoradiotherapy in esophageal cancer (4) or adjuvant chemotherapy in colon cancer (5). However, there are only a few scenarios in which chemotherapy results in robust and durable cures for metastatic solid cancers, with testicular cancer being the most important example (6–8). In almost all other metastatic cancers, clinical responses to systemic chemotherapy are partial at best, and then only in a subset of patients (**Figure 1**). This variability in chemo‐responsiveness occurs not only between patients with different tumor types, but also within groups of patients with the same tumor type. For example, in patients with esophageal cancer treated with carboplatin/paclitaxel in combination with radiotherapy, 30% have a histologically confirmed complete regression of their tumor, while 20% display no clinical response (4). Similarly, in early stage testicular cancer, adjuvant chemotherapy is curative and induces a robust clinical response in all but a small subset of patients (8). Also, in settings where chemotherapy rarely, if ever, results in complete regression, such as in mesothelioma, responses are still diverse, with approximately 40% of patients displaying an objective clinical response (12). This heterogeneity in response between patients with the same cancer type is not well understood. Given the frequent and sometimes severe toxicity of many chemotherapeutics, weighed against a beneficial response in only a subset of patients, there remains an urgent need for predictive biomarkers. However, despite many attempts, there are no robust and validated pre-treatment biomarkers that can guide clinical decision making.

In this review, we explore both the cell intrinsic (factors at the individual cell level) and cell extrinsic (factors within the tumor microenvironment) drivers of chemotherapy sensitivity or resistance. We then summarize the literature regarding the relationship between proliferation rate and chemotherapy sensitivity. Lastly, we describe the components of the tumor microenvironment and the roles they play in chemotherapy efficacy and propose a model of a chemo-sensitive tumor.

## Mechanisms of inherent individual cell sensitivity and resistance to cytotoxic chemotherapy

**Figure 2:**
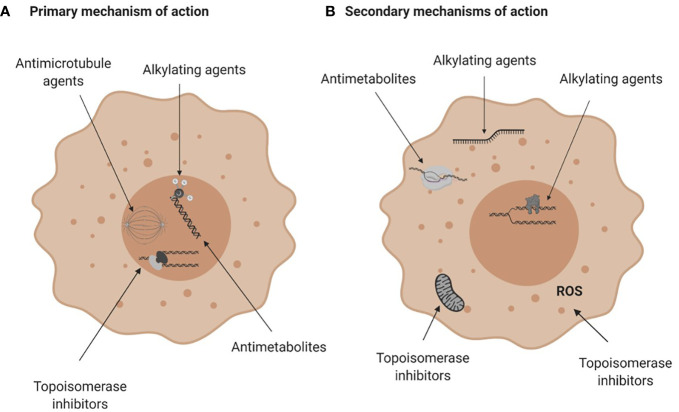
Mechanisms of action of conventional chemotherapies. **(A)** Primary mechanisms of action. Alkylating agents induce DNA breaks, anti-metabolites are incorporated into DNA or RNA and interfere with DNA and RNA synthesis, topoisomerase (Top) inhibitors damage the Top I or Top II enzymes halting DNA replication and anti-microtubule agents damage microtubules and affect mitosis. **(B)** Secondary mechanisms of action of chemotherapies. Alkylating agents can bind to RNA or induce protein-DNA crosslinks, antimetabolites can inhibit enzymes crucial for DNA or RNA synthesis and topoisomerase inhibitors can impair mitochondria biogenesis or generate reactive oxygen species. For more see **Table 1**. Figure created with BioRender.com.

Conventional chemotherapies are divided into several classes based on their primary or ostensible mechanism of action. They include alkylating agents and platinum analogues, which induce inter- or intra- strand DNA crosslinks that destabilize DNA and cause DNA breakage; antimetabolites that inhibit the synthesis of DNA, RNA or their components; topoisomerase inhibitors that block the DNA unwinding enzymes; and microtubular poisons that act on tubulin, impeding the mitotic spindle and stalling cell division (**Table 1**; **Figure 2A**). These drugs also have known secondary mechanisms of action, such as effects on mitochondrial biogenesis (22) or the production of reactive oxygen species (32), which contributes to their cytotoxicity (**Figure 2B**).

**Table 1:** Mechanism of action of classic chemotherapies.

Chemotherapy class	Examples	Primary mechanism of action	Additional mechanism of action
Antimicrotubule agents	Taxanes (paclitaxel, doxorubicin)	Binding to interior surface of microtubules, impeding movement and function (17)	Altering of cell signaling and trafficking, slowing of cell cycle progression, inhibiting cell migration and invasiveness, disrupting tumor vasculature (18)
	Vinca alkaloids (vinblastine, vincristine, vinorelbine)	Depolymerizing microtubules, destroying mitotic spindles at high concentrations and blocking mitosis at low concentrations (19)	
Topoisomerase (Top) inhibitors	Camptothecin analogues (irinotecan and topotecan), anthracyclines (doxorubicin and daunorubicin and their derivatives epirubicin and idarubicin), mitoxantrone, dactinomycin, etoposide and teniposide	Binding to Top by intercalating DNA to create a drug/enzyme complex. When the replication fork reaches this complex the collision causes double stranded DNA breaks (20)	Generation of oxygen free radicals (21). Targeting of Top 2β to impair mitochondrial biogenesis and inducing cell death in non-proliferating cells (22).
Alkylating agents	cyclophosphamide, mitomycin, dacarbazine, procarbazine, temozolomide and streptozocin	Inducing DNA damage by transferring alkyl groups to DNA, generating covalent adducts that induce single or double stranded DNA breaks (23)	Affect RNA, proteins, lipids and mitochondrial DNA (24), generate additional toxic products and mutagenic lesions (23) Generation of reactive oxygen species (25)
	anthracyclines (doxorubicin and daunorubicin and their derivatives epirubicin and idarubicin)*	Intercalating with DNA (26, 27)	
	Platinum based chemotherapies (Cisplatin, carboplatin and oxaliplatin)	Forming inter-,or intra-strand DNA crosslinks that induce DNA damage and interfere with DNA repair, DNA replication and DNA transcription (28)	Affect RNA and proteins, generate DNA-protein crosslinks (29). Generation of reactive oxygen species (30)
Antimetabolites	5‐Flurouracil (5‐FU), cytarabine, gemcitabine, the 6-thiopruines (comprising of 6‐mercaptopurine and 6-thioguanine) and clofarabine	Incorporated into DNA instead of regular nucleotides or molecules, which inhibits of DNA synthesis and causes premature chain termination (23) Gemcitabine, cytarabine and fludarabine also inhibit DNA polymerase and ribonucleotide reductase to halt DNA replication, chain elongation and DNA repair (31)	

*Anthracyclines can be classed as both alkylating agents and topoisomerase inhibitors.

Cancer cells experience different fates after drug exposure; some cells are killed while others escape cell death and survive (33, 34). Factors that contribute to the induction of apoptosis can act before DNA damage occurs (at the level of uptake of drug into the cell and the efflux of the drug out of the cell or the metabolism of drug to active metabolites); be associated with DNA damage (at the level of drug binding to target molecules, altered DNA repair enzymes or tolerance to DNA damage); or act after DNA damage has occurred (due to altered sensitivity to apoptosis, altered cell signaling or stochastic effects) and vary depending on the mechanism of action of the chemotherapy. Despite our increased understanding of these mechanisms of sensitivity and resistance this has not translated to the clinical implementation of a predictive biomarker of response to chemotherapy, nor the widespread use of combination therapies that exploit these pathways to improve drug effectiveness.

### Uptake and efflux of chemotherapy drugs

While it is perhaps not surprising that cellular chemotherapy uptake varies between patients and tumor types, large differences in uptake are also observed *in vitro* between cells within the same clonal culture, resulting in differential therapeutic sensitivity (35, 36). Cellular features that can modulate intracellular levels of chemotherapy include efflux pumps which have been implicated in chemotherapy resistance (37–39). These drug efflux pumps, most notably p-glycoprotein, impede the transportation of chemotherapy into the cell. There have been numerous studies over the last few decades demonstrating that inhibition of these pumps improves chemotherapy uptake and tumor sensitivity *in vitro* (40–42) and *in vivo* (43–45).

### Genetic variations

Cancer cells can harbor intrinsic mutations that render them less sensitive to chemotherapy. It is important to distinguish these inherent mutations that are present before treatment, from acquired mutations that the cells gain after treatment which provide a selective survival advantage (46, 47). For example, the dysregulation of components of the apoptotic pathway that enhance survival can increase drug resistance (48). Mutations in TP53, a key tumor suppressor, are associated with resistance to DNA damage induced by chemotherapy (49–52). Abnormalities in another tumor suppressor commonly dysregulated in cancer, retinoblastoma protein, is also associated with chemotherapy response in patients with lung cancer (53), breast cancer (54, 55), non-small cell lung cancer (56), and colorectal cancer (57) with the absence of retinoblastoma protein correlating with improved survival. Intrinsic mutations in the components of the apoptotic pathway are also associated with reduced sensitivity to chemotherapy. Lastly, genetic variations can occur in the proteins that some chemotherapies, primarily antimetabolites, target. For example, methotrexate binds to the enzyme DHFR to execute its anti-tumor effect and mutations in DHFR can alter chemo-sensitivity (58, 59).

### Altered DNA damage repair pathways

As highlighted, a key mechanism of action of many chemotherapies is the induction of DNA damage which leads to the activation of cell death pathways. Acting against DNA damage are multiple repair pathways; base excision repair, mismatch repair, homologous recombination and non-homologous end-joining (60). Increased expression of nucleotide excision repair related genes correlates with resistance to platinum-based drugs. For example high expression of the excision repair cross-complementation group 1 factor is a known mechanism for cisplatin resistance in numerous cancers (61). Another example is ribonucleotide reductase subunit M1 which converts ribonucleotides into the deoxyribonucleotides required for DNA replication and DNA repair (62) and is inhibited by gemcitabine. Expression of RRM1 is inversely correlated with survival and sensitivity to platinum-based chemotherapy and gemcitabine (63, 64) in lung cancer or pancreatic cancer patients, though these findings vary with other studies finding no association with survival (65, 66).

### Cell cycle

The cell cycle is intrinsically linked to chemotherapy efficacy because the primary mechanism of action of many drugs is to affect components crucial to cell division such as DNA replication or the formation of mitotic spindles. The cell cycle specificity of chemotherapies has been demonstrated *in vitro*. In the case of anti-mitotic chemotherapies, cytotoxicity is rarely induced until the cell enters mitosis, where it is most vulnerable (34). *In vivo* models to assess the effects of drugs on cell cycle often utilize the Fluorescent Ubiquitination-based Cell Cycle Indicator (FUCCI) system, in which cells express different fluorescent proteins as they progress throughout the cell cycle (67). In mice with orthotopic human gastric cancers, 68% of the cells were in S, G2 or M phase before treatment and 32% in G1 or G0 (68). After treatment with cisplatin or paclitaxel, more than 90% were in G1 or G0, indicating that the cells actively undergoing proliferation (those in S, G2 or M) were selectively targeted by the drugs. When cancer cells are treated with *Salmonella Typhimurium AR-1* or recombinant methionine, trapping the cells in S/G2, they become more sensitive to subsequent treatment with cisplatin or paclitaxel, further demonstrating the cell cycle specificity of chemotherapy (69).

### Cancer stem cells

Cancer stem cells (CSCs) are a small population of cancer cells with the capability of self-renewal and have high tumorigenic and metastatic potential (70). CSCs can be inherently resistant to chemotherapy due to a multitude of factors such as their slow proliferation rate and quiescent nature (71), active anti-apoptotic machinery (72, 73), efficient DNA repair systems (74, 75), effective modulation of reactive oxygen species (76) and their robust and stable expression of drug efflux pumps (77).

### Chemotherapy induced senescence

A wide range of chemotherapies spanning most of the classes have been found to induce senescence both *in vivo* and in patient samples collected after treatment (78). These cells remain viable and metabolically active but are unable to proliferate. The induction of senescence by chemotherapy could be both beneficial and harmful to patient outcomes. As these cells do not divide and remain arrested in G1 or G2/M and can remain in this dormant state for an extended period of time, there is some degree of disease control (79, 80). However, the induction of senescence can be incomplete and is reversable, with the treatment resistant clones escaping cell cycle arrest and inducing disease relapse (81).

### Stochastic differences affecting chemotherapy sensitivity

Lastly, the sensitivity of individual cancer cells to chemotherapy may differ due to stochastic differences. *In vitro* studies using genetically identical clonal cell lines, exposed to identical drug levels and corrected for cell cycle, found that there were significant differences in chemotherapy sensitivity between individual cells (33, 34, 82). This highlights that seemingly identical cancer cells differ in their chemo-sensitivity, even when all external factors are controlled for. Cancer cells exist in an equilibrium of pro- and anti- apoptotic proteins, where an additional stimulus can easily induce apoptosis. These cancer cells are termed ‘primed’ for apoptosis (83, 84) and are more chemo-sensitive than ‘un-primed’ cells (85). Heterogeneity in the chemotherapy response can be attributed to variability in the expression of key proteins, whereby some cells are primed for apoptosis and have a lower threshold of stimuli for the activation of cell death pathways due to the up- or down-regulation of specific pathways. For example, multiple myeloma is characterized by the overexpression of the anti-apoptotic proteins Bcl‐2 or Mcl-1 which favors cancer cell resistance to chemotherapy (86).

The influence of stochastic differences in chemotherapy sensitivity is further demonstrated in the observation that there is a moderate level of cell-cell variability in protein abundance in untreated cells and only 20% of this variability can be attributed to differences in cell cycle stage (33). When protein levels were measured before and after chemotherapy treatment, most of the profiles were similar before and after chemotherapy exposure in each individual cell. Interestingly, there was a small subset of proteins that displayed bimodal behavior, with increased levels in a subset of cells and decreased levels in others. Two of these proteins showed behavior that correlated with cell fate, indicating that the stochastic differences in protein expression between cells may contribute to the ability to escape chemotherapy induced cell death. These studies also demonstrated that the fate of individual sister cells can be independent from each other (34) and that individual subclones exhibit heterogeneity in the response to chemotherapy (34, 82), further highlighting the role of stochastically driven heterogeneity in the chemosensitivity of cancer cells.

### Approaches to target cellular mechanisms of resistance to improve chemotherapy efficacy

Since the identification of the mechanisms of inherent cellular resistance to chemotherapy, novel drugs have been developed to target and inhibit drivers of resistance to improve chemotherapy efficacy. Targeting DNA repair pathways using poly(ADP-ribose) polymerases (PARP) inhibitors is one avenue that has shown promise. As PARP acts to recruit DNA repair proteins to promote repair of DNA breaks as well as homologous recombination, the inhibition of PARP limits DNA repair after damage which could augment the effects of chemotherapies that damage DNA (87). The addition of PARP inhibitors to chemotherapy have shown some clinical efficacy (**Table 2**) and work is ongoing to expand these findings to other cancers and chemotherapy combinations (107).

**Table 2:** Clinical studies that combine chemotherapy with agents that target cellular mechanisms of chemo-resistance. PARP, poly(ADP ribose) polymerase.

Mechanism of resistance	Molecular target	Drug	Disease	Efficacy of combination with chemotherapy compared to chemotherapy alone
Drug efflux pumps	p-glycoprotein	Verapamil	Non-small cell lung cancer	Improved survival (88)
			Small cell lung cancer	No improvement in survival or response rate (89)
			Ovarian cancer	No improvement in response rate and significant toxicity (90)
		Quinine	Acute myeloid leukemia	No improvement in overall survival (91)
		Dofequidar	Breast cancer	No significant improvement in survival or response rate (92)
DNA repair pathways	Poly (ADP ribose) polymerase (PARP) inhibitors	Rucaparbid	Ovarian cancer	Improved progression free survival in patients who responded to initial treatment (93)
		Veliparib	BRCA+ Ovarian cancer	No improved response rate or progression free survival (94)
		Olaparib	Ovarian cancer	Improved progression free survival but no improvement in overall survival (95)
		Olaparib	Gastric cancer	No improvement in overall survival (96)
Anti-apoptotic proteins	Bcl-2 inhibition or decrease in expression	12-cis retinoic acid and IFNa	Prostate cancer	Indications of clinical activity (97)
		Navitoclax	Solid cancers	Tolerated, did not compare to placebo (98)
		Venetoclax	Acute myeloid leukemia	Improved overall survival (99)
				No improvement in overall survival, increased rate of remission and increased duration of response (100).
			Chronic lymphocytic leukemia	Tolerated, did not compare to placebo (101).
			Multiple myeloma	Tolerated, did not compare to placebo (102).
Inhibition of CSC signaling pathways	Notch2/3	Tarextumab	Pancreatic cancer	No improvement in overall survival (103)
	WNT signaling	Ipafricept	Ovarian cancer	Toxicity (104)
		Ipafricept	Pancreatic cancer	Toxicity (105)
		Vantictumab	Breast cancer	Toxicity (106)

However, the expansion of this strategy to other pathways or drugs has proven difficult with mixed results from clinical trials and the discontinuation of studies due to excessive toxicity (**Table 2**). One example relates to drugs that target drug efflux pumps, particularly those mediated by p-glycoprotein. Clinical trials have not found a significant survival benefit using combination therapy with drug efflux pump inhibitors and chemotherapy (89–91) or only a slight improvement in a subset of patients (88), and development has been hindered by the levels of toxicity associated with the dose required for a clinical benefit to be achieved. Similarly, some drugs targeting the WNT signaling pathway which is important in both conventional stem cells and CSCs (108) have had to be discontinued due to toxicity, primarily in the bone marrow leading to increased incidence of fractures (105). Lastly, drugs that inhibit or decrease the expression of the anti-apoptotic protein Bcl-2 are well tolerated and induce substantial responses when used as a monotherapy or combined with dexamethasone in chronic lymphocytic leukemia or multiple myeloma (101, 102) or combined with azacytidine, decitabine or low-dose cytarabine in acute myeloid leukemia (99), the latter receiving FDA approval.

## Proliferation and chemotherapy sensitivity-the proliferation rate hypothesis

An underlying commonality between the classes of chemotherapeutics discussed in this review is that their mechanism of action primarily affects biological processes associated with cell division, either by inducing DNA damage or by directly inhibiting mitotic progression. Since a key hallmark of cancer is rapid and uncontrolled cellular division, cancer cells are therefore thought to be more sensitive to chemotherapeutic drugs then normal tissues. This has led to the general acceptance within the biomedical community that the cancer specificity of chemotherapy comes from the preferential killing of rapidly proliferating cells (109). However, the experimental results that support this notion are relatively limited and mixed.

Firstly, the relationship between proliferation rates and chemo-sensitivity *in vitro* is not straightforward. For example, Kondoh et al. analyzed the correlation between doubling time and sensitivity of anticancer drugs against the NCI-60 panel of cancer cell lines (110). Although the authors found that majority of anticancer drugs had higher efficacy in faster dividing cell lines, this was not the case for all chemotherapeutics and not for all cancer cell lines. These conflicting and varying results are also evident from other studies (41, 111), including studies in which the proliferation rate was modulated, through either pharmacological means or gene silencing, showing varying degrees of both decreased (110, 112) and increased sensitivity to chemotherapy (113–116).

**Figure 3:**
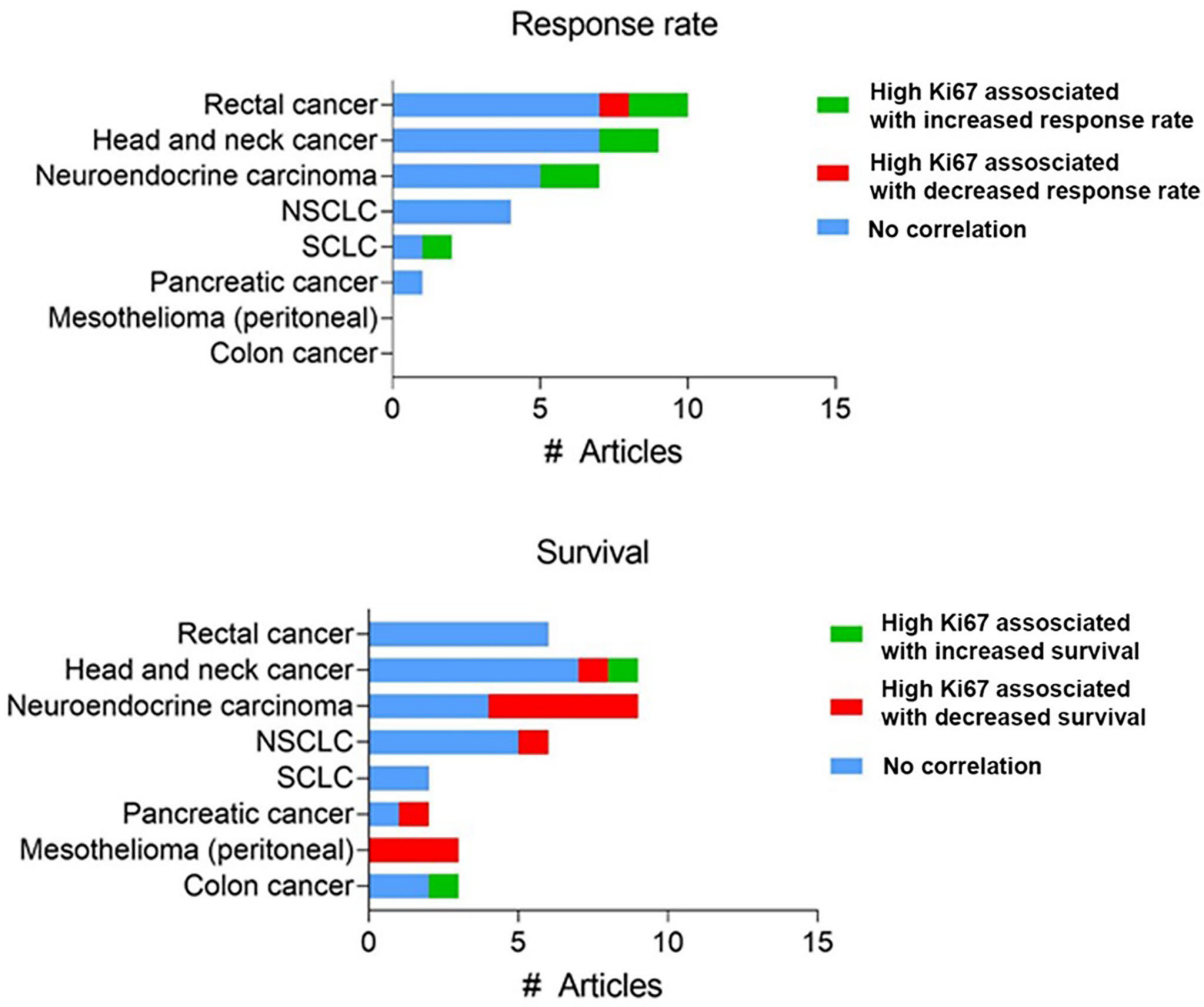
Reported clinical studies testing a correlation between Ki67 expression and chemotherapy response. Number of studies assessing Ki67 expression using immunohistochemistry with reported correlation between Ki67 expression and response rate or survival. Full dataset in **Supplementary Table 1**.

Secondly, there is limited *in vivo* validation of the increased effect of chemotherapy in highly proliferating tumors. Many studies measured proliferation at either a fixed timepoint within the tumor or used *in vitro* rates of cell division and correlated this with *in vivo* response. For example, Nakasone et al. demonstrated that the *in vivo* difference in sensitivity between different tumor models could not be attributed to differences in *in vitro* proliferation rate (117). The development of intravital fluorescent imaging, utilizing the FUCCI system (67) has made it possible to assess proliferation over time within the tumor itself, overcoming the caveats of previous studies. Yano et al. utilized these methods to monitor the cell cycle progression in an orthotopic model of liver cancer during chemotherapy treatment (68). When tumors with most cells in S/G2/M phase (an actively cycling or proliferating tumor) cisplatin or paclitaxel treatment resulted in significant cancer cytotoxicity, while there was little anti-tumor effect when cells were mainly in G0/G1 (69). Although these data suggest a correlation between cell cycle stage (and by extension proliferation rate), clinical data to substantiate this hypothesis are limited and mixed; the use of proliferation rate as a biomarker for response to chemotherapy varies greatly between cancers and is limited in its predictive power. For example, there is a striking absence of any significant and reproducible correlation between high expression of Ki67 and chemotherapy response in many cancer types (**Figure 3**). A systematic review in breast cancer found a correlation between Ki67 expression before neoadjuvant chemotherapy and overall/progression-free survival in 10/20 and 17/33 studies, respectively (118). Similarly, one meta-analysis found that high (>10%) Ki67 positivity is associated with decreased survival (119), while another reported that high Ki67 could predict response and clinical benefit from neoadjuvant chemotherapy (120).

For these reasons, others have previously critically challenged the assumption that chemotherapy particularly targets cancer cells because they are rapidly proliferating (18, 109, 121). Regardless, the inability to consistently correlate cell proliferation rate with chemotherapy response in patients highlights that there are likely additional drivers of chemotherapy sensitivity.

## The tumor microenvironment: the driver of chemotherapy sensitivity

### Stroma and vasculature: a role in chemotherapy resistance

**Figure 4:**
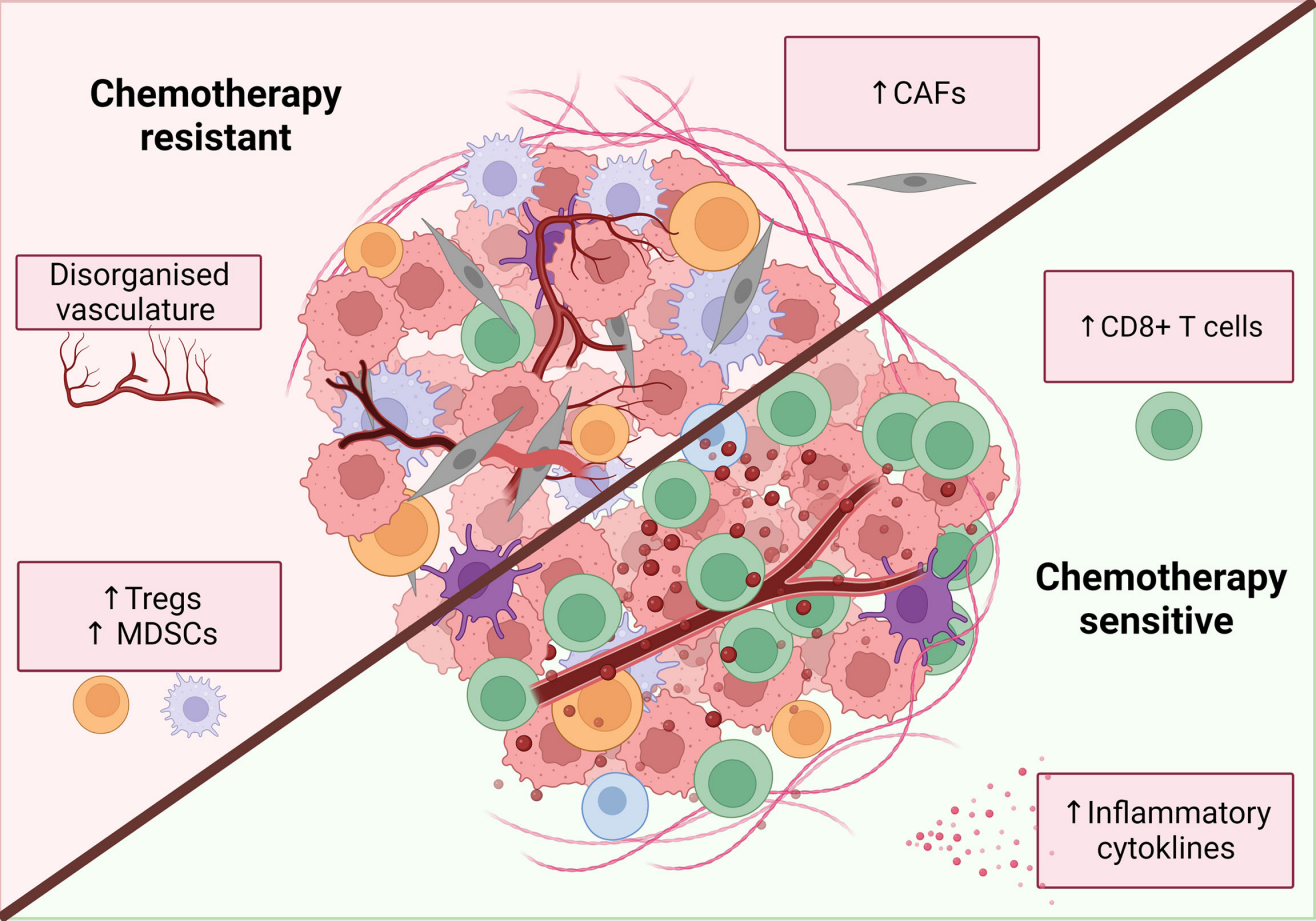
Characteristics of a chemotherapy sensitive TME. An inflammatory, immune infiltrated ‘hot’ tumor is associated with response to classical chemotherapies. These tumors are characterized by the infiltration of immune cells, particularly increased CD8^+^ T cells. release of inflammatory mediators such as IFNs and TNFα and decreased levels of immunosuppressive cells. Additionally, tumor vasculature can have both positive and negative effects on chemotherapy response while CAFs are primarily associated with chemotherapy resistance. Figure created with BioRender.com.

A tumor is a complex and dynamic environment of immune cells, extracellular matrix, fibroblasts and vasculature which make up the tumor stroma, which can all influence chemotherapy sensitivity (**Figure 4**).

Cancer associated fibroblasts (CAFs) are one of the key components of the tumor stroma that have been implicated in tumor progression and resistance to chemotherapy. CAFs are phenotypically different from other fibroblasts and secrete cancer-promoting factors including TGF‐β, vascular endothelial growth factor, platelet-derived growth factor and fibroblast growth factor 2 (122). The secretion of these factors are associated with an enhancement in the invasive and metastatic ability of cancer cells (123, 124), stimulation of angiogenesis (125, 126),and expression of anti-apoptotic proteins (127) and promote an immunosuppressive tumor microenvironment (128) which modify the TME to support tumor cell survival and chemotherapy resistance (129). *In vitro* studies have confirmed that these soluble factors induce chemotherapy resistance (130–133) and targeting of CAFs *in vivo* enhances the anti-tumor effects of chemotherapy in animal models (134–136). CAFs also contribute to desmoplasia which is the formation of fibrotic tissue around tumor cells. This creates not only a physical barrier around the tumor, limiting the penetration of drugs, but also can increases interstitial pressure which compresses blood vessels, also decreasing drug availability within the TME (137, 138). Clinical studies found high levels of CAFs were associated with poor PFS following chemotherapy (139–142).

As chemotherapy is typically administered systemically, it is essential that the drug reaches the tumor at a sufficient concentration. Therefore, tumor vasculature is not only essential for cancer growth, but also for the distribution of chemotherapy to the tumor. Patients with tumors exhibiting a lower density of blood vessels indeed have a poorer response to chemotherapy (143, 144). However, tumor vasculature is often disorganized and characterized by neo-angiogenesis and the modification of existing vessels within the tissue stroma which can impede the distribution of chemotherapy. Strategies to ‘normalize’ tumor vasculature and reverse the dysfunctional structure have proven to be effective in murine studies (145–148). When drugs targeting tumor vasculature are combined with chemotherapy, both the chemotherapeutic dose reaching the tumor and subsequent anti-tumor response is more effective compared to chemotherapy alone (145, 146, 149). Clinical studies have demonstrated primarily positive results. For example, the addition of bevacizumab (a VEGF-A inhibitor) to chemotherapy regimens lead to an increase in both progression free and overall survival in mesothelioma (150) and ovarian cancer (151, 152). No clinical benefit was seen when combined with chemotherapy in early stage NSCLC (153) while in colon cancer bevacizumab improved survival in the metastatic but not adjuvant setting (154, 155).

### Immunity, inflammation and chemotherapy efficacy

Spurred on by the emergence and success of immunotherapy, the role of the immune system within the tumor stroma is now a key focus of research into chemotherapy efficacy. However, because chemotherapy is leukodepleting, it was historically thought to be predominantly immunosuppressive. The important role of immune cells in the efficacy of chemotherapy has been highlighted in recent years, first demonstrated by Schwartz in 1973 (156) and more recently driven by the work of Nowak (157, 158) and Zitvogel and Kroemer (159, 160). The latter group compared the response to chemotherapy in immunodeficient (predominantly either Nu/Nu or Rag1-/- mice which lack functional T cells and functional T and B cells respectively) and immunocompetent mice (159, 161). Chemotherapy was significantly less effective in mice lacking an intact immune system, while these drugs were extremely effective in the wildtype counterparts. The requirement of an intact immune system for chemotherapy to induce an effective anti-tumor response has been tested using numerous cancer models and chemotherapies (**Table 3**). Genetic mouse models did not provide the same convincing results as transplantable models, which tend to be more immunogenic (165).

**Table 3:** Effect of chemotherapy in immunodeficient Nude/Rag mice compared to immunocompetent wildtype (WT) mice.

Chemotherapy	Cell Line	Effect in Nude/Rag compared to WT	Ref
Oxaliplatin	EL4 Lymphoma	Decreased	(162)
	CT26	Slight decrease	(163, 164)
	MMTV-NeuT *	No difference	(165)
	K14cre;CdhI^flox/flox^;Trp53^flox/flox^ *	No difference	(165)
	GOS	Decreased	(166)
Mitoxantrone	CT26	Decreased	(161, 163)
	MCA205	Decreased	(163)
Cisplatin	CT26	Decreased	(164)
	MMTV-NeuT *	No difference	(165)
	K14cre;CdhI^flox/flox^;Trp53^flox/flox^ *	No difference	(165)
Doxorubicin	MMTV-NeuT	No difference	(165)
	CT26	Decreased	(159)
Cyclophosphamide	AB1-HA	Decreased	(167)
Etoposide	Eu-MYC	No difference	(168)
Irinotecan	GOS	No difference	(166)
Docetaxel	PO3	No difference	(166)
Gemcitabine	TC-1	Decreased	(169)
	AB12	Decreased	
	EJ-6-2	Decreased	
Cyclophosphamide/gemcitabine	CT26	Decreased	(170)
Oxaliplatin/cyclophosphamide	KP NSLC	Decreased	(171)

Effects are reported as decreased (effiacy decreased in immunodeficient mouse), no difference (efficacy the same in immunodefcient and wildtype mice) or increased (efficacy is increased in immunodeficient mouse). *genetically engineered mouse cancer model

Studies using depleting antibodies against specific immune cells and cytokines or the knockout of specific genes or cellular receptors have helped to define the cell types and pathways that play an important role in the chemotherapy response (**Tables 4**, **5**). Experiments where IFNγ was neutralized using knock‐out mouse models found that the absence of signaling decreased chemotherapy efficacy *in vivo* (162, 167, 173, 174). The idea of an immunostimulatory ‘hot’ TME has been popularized in the context of immunotherapy: highly immune infiltrated, inflammatory tumors with increased expression of IFN-related genes are associated with improved response to immune checkpoint therapy (179–181). Indeed, several studies suggest an inflammatory, immune infiltrated TME is associated with chemotherapy sensitivity, with complete responders to chemotherapy having increased immune infiltration (178, 182–184). In particular, responders to chemotherapy have increased levels of CD8^+^ T cells (182, 185, 186) and the upregulation of IFN related genes (178, 182). The presence of immunosuppressive cells like regulatory T cells (Treg) or myeloid suppressor cells (MDSC) are associated with a decreased response to chemotherapy, presumably due to the promotion of an immunosuppressive TME (187–191). The contribution of other immune cells to chemotherapy sensitivity is less well characterized and may vary depending on drug or tumor model (**Table 3**) and further studies are required to fully elucidate their role in chemotherapy‐driven anti-tumor immunity.

**Table 4:** Effect of chemotherapy in immune cell depleted mice compared to immunocompetent wildtype mice.

Depletion	Chemotherapy	Cell Line	Effect on response compared to WT	Ref
CD8^+^ T Cells	Oxaliplatin	EL4	Decreased	(162)
		K14cre;CdhI^flox/flox^;Trp53^flox/flox^ *	No difference	(165)
	Cisplatin	TC-1	Decreased	(172)
		C3	Decreased	(172)
	Doxorubicin	AT3	Decreased	(173)
		H2N100	Decreased	(173)
		EO771	Decreased	(173)
		MCA205	Decreased	(173)
		MCA2	Decreased	(173)
		CT26	Decreased	(159)
	Cyclophosphamide	CT26	Decreased	(174)
		AB1-HA	Decreased	(167, 175)
	Paclitaxel	RENCA +	Decreased	(176)
	Oxaliplatin/cyclophosphamide	KP	Decreased	(171)
CD4^+^ T Cells	Cisplatin	TC-1	No difference	(172)
	Cyclophosphamide	CT26	No difference	(174)
		AB1-HA	No difference	(175)
	Paclitaxel	RENCA +	Increased	(176)
NK Cells	Cisplatin	TC-1	No difference	(172)
	Doxorubicin	CT26	Decreased	(159)
	Cyclophosphamide	CT26	No difference	(174)
DC and Macrophages	Cisplatin	TC-1	Slight decrease	(172)
B cells	Doxorubicin	MMTV-pyMT	Decreased	(177)
	Doxorubicin	4T1	Slight decrease	
	Cisplatin	4T1	Slight decrease	
Tregs (αCD25)	Cyclophosphamide	CT26	No difference	(174)
	Paclitaxel	RENCA	No difference	(176)

Effects are reported as decreased (effiacy decreased in immune depleted mouse), no difference (efficacy the same in immune cell depleted and wildtype mice) or increased (efficacy is increased in immune cell depleted mouse). *genetically engineered mouse cancer model +metastatic tumor model

**Table 5:** Effect of chemotherapy in knock out mice compared to immunocompetent wildtype mice.

Knockout	Depletion	Chemotherapy	Cell Line	Effect in K/O compared to WT	Ref
IFNγ -/-	IFNγ	Oxaliplatin	EL4	Decreased	(162)
		Oxaliplatin	EG7	Decreased	(162)
		Cyclophosphamide	AB1-HA	Decreased	(167)
		Cyclophosphamide	CT26	Decreased	(174)
		Doxorubicin	E0771	Decreased	(173)
IL12RB2-/-	IL12 Receptor	Oxaliplatin	EL4	No change	(162)
Tnfsr10-/-	TNF Receptor	Oxaliplatin	EL4	No change	(162)
Prf1-/-	Perforin	Oxaliplatin	EL4	No change	(162)
Pfp-/-	Perforin	Cyclophosphamide	AB1-HA	No change	(167)
IFNyR1-/-	IFNγ Receptor	Oxaliplatin	EL4	Decreased	(162)
		Oxaliplatin	EG7	Decreased	(162)
P2RX_7_-/-		Oxaliplatin	EL4	Decreased	(162)
NLRP3-/-		Oxaliplatin	EL4	Decreased	(162)
CASP1-/-	Caspase 1	Oxaliplatin	El4	Decreased	(162)
Jh-/-	B cells	Doxorubicin	E0771	Decreased	(177)
		Docetaxel	E0771	Decreased	
TRAIL -/–		Cyclophosphamide	AB1-HA	Decreased	(167)
Tlr4-/-		Cisplatin	TC-1	No change	(172)
CD80/CD86-/-		Cisplatin	TC-1	Decreased	(172)
CD70/CD80/CD86-/-		Cisplatin	TC-1	Decreased	(172)
IL-1B -/-		Doxorubicin	AT3	Decreased	(173)
			E0771	Decreased	(173)
IL17A -/-		Doxorubicin	AT3	Decreased	(173)
			E0771	Decreased	(173)
IL-23p19 -/-		Doxorubicin		No Change	(173)
TCRJα18	NKT	Doxorubicin	MCA205	No Change	(173)
			AT3	No Change	(173)
TCRδ -/-	Γδ T Cells	Doxorubicin	MCA205	Decreased	(173)
			AT3	Decreased	(173)
Anti-IFNAR		Doxorubicin	MCA205	Decreased	(178)

Effects are reported as decreased (effiacy decreased in knock-out mice), no change (efficacy the same in knock-out and wildtype mice) or increased (efficacy is increased in knock-out mice).

It is evident from the above studies in mouse models and patients that the composition of the TME plays a critical role in chemotherapy efficacy. It must also be noted that chemotherapy has numerous positive and negative effects on immune cells, which have been reviewed in detail previously (160, 192). The fact that chemotherapy is one of the most efficacious combinatorial treatments with immune checkpoint therapy, suggests at the very least that chemotherapy treatment is not an immunological null-event, and that its beneficial immunological effects can be exploited therapeutically (3, 193, 194).

### Tracking dynamic changes in the tumor microenvironment correlating with treatment outcome

Increased understanding of the role of the TME in the response to chemotherapy has led to a large body of work on identifying predictive biomarkers from the TME. A detailed understanding of the effects of chemotherapy on the various components of the TME would help with the selection of cell populations, genes or proteins for use as a predictive biomarker. While there has been a significant amount of work in this field, there has been little progress of integrating the use of pre-treatment biomarkers into routine use within the clinic that could predict response. It must be noted that whether a patient achieves a pathological complete response when chemotherapy is used in a neoadjuvant setting is a robust indicator of clinical outcome, as for most cancers, these patients have increased disease-free survival and overall survival compared to patients with residual disease at surgery (195).

One of the limitations of the most common approach to biomarker discovery is that tumor or blood samples are only collected at a single timepoint, usually before treatment. This only gives a ‘snapshot’ of the tumor microenvironment, or the systemic environment in the case of blood sample. Taking serial samples would allow the effects of chemotherapy to be monitored throughout therapy. Whether a patient is responding or not would be able to be determined earlier, allowing physicians to make a more informed decision on whether to continue with the current treatment or not. Moreover, it would allow a deeper understanding of the biological mechanisms that are responsible for an effective chemotherapy response, allowing the development of novel rational combination therapies. Measuring the changes within the tumor that are induced by chemotherapy is hindered by the inability to obtain serial tumor samples from patients throughout the course of their therapy, primarily due to the location of the cancer and invasive procedures required to retrieve a biopsy. Often clinical studies use peripheral blood (196, 197) or effusions as a surrogate for the tumor microenvironment, however it is not clear whether these samples provide a meaningful representation of the events occurring within the tumor itself (198). Studies that do examine the changes within the TME during therapy differ in the parameters measured, often only measuring a selection of markers, making it difficult to compare between studies, and likely resulting in an incomplete representation of what happens throughout the whole TME.

The few studies that investigated changes in the TME during chemotherapy and correlated these changes with clinical response highlight the importance of serial measurements instead of pre- or post- treatment snapshots. Many studies found no difference between responders and non-responders when baseline levels of their chosen markers were compared (**Supplementary Table 2**). However, when the change in expression from pre-treatment to post-treatment was interrogated, the differences between responders and non-responders became apparent (199–202). Molecules involved in chemotherapy resistance, for example GSTP1 (an enzyme associated with decreased sensitivity to cytotoxic agents including anthracyclines (203)) or ALDH1 (an enzyme involved in detoxifying aldehydes into weaker metabolites), decrease in patients whose tumors respond to chemotherapy (200, 204). Decreased GSTP1 expression after chemotherapy, is more prominent in tumors of patients with breast cancer that respond to doxorubicin and cyclophosphamide chemotherapy and is associated with improved progression free survival (204). Similarly, decreased tumor expression of biomarkers associated with tumorigenesis (COX-2) or immune evasion (PD-L1) is noted throughout treatment, with the decrease more prominent in responders (199, 205). The primary limitation of these studies is that they only assessed a small number of markers or cell populations, using immunohistochemistry or flow cytometry. This makes it difficult to capture the complexity of the TME. The increasing ability to obtain high dimensional biological data using for example single cell RNAseq or spatial transcriptomics provides an avenue for a deeper characterization of the TME during chemotherapy treatment.

## Summary and key outstanding questions

Based on the available data, an incomplete picture emerges of a chemotherapy-sensitive TME, which includes CD8 T cell infiltration, activation of inflammatory pathways such as IFNs, low levels of CAFs and a normalized vasculature (**Figure 4**).

An added complexity is the wide range of chemotherapeutics used in the clinic, spanning different classes with vastly different mechanisms of action and immune effects. Uncovering what drives chemotherapy efficacy opens the door to the development of predictive biomarkers and novel combination treatments. While immune checkpoint therapy has shown promise in a multitude of cancer types, like chemotherapy, it is not effective in all patients. Having a predictive biomarker for a robust response to chemotherapy, either on its own or in combination with immunotherapy, would significantly improve the potential of clinical decision making, allowing patients to stratified based on their likelihood of a beneficial response to either treatment.

An additional question is whether the TME can be modulated and transformed from chemotherapy-resistant to chemotherapy-sensitive. Pre-treating a patient to induce a sensitive TME phenotype has improved the response to immunotherapy in preclinical models (206, 207). The dependence of chemotherapy efficacy on the immune system and early indications of synergy between chemotherapy and immune checkpoint therapy (208–210) highlights the opportunity to alter the tumor immune milieu to improve the anti-tumor immune response generated by chemotherapeutics.

## Author contributions

CT wrote the article and generated the figures and tables. SF, RL, AN, and WL critically revised the manuscript. All authors contributed to the article and approved the submitted version.

## Funding

WL was supported by fellowships from the Simon Lee Foundation, NHMRC (grant names APP1126076 and APP1196605) and Cancer Council WA. The National Centre for Asbestos Related Diseases received funding through the NHMRC Centre of Research Excellence scheme (grant number APP1197652).

## Conflict of interest

The authors declare that the research was conducted in the absence of any commercial or financial relationships that could be construed as a potential conflict of interest.

## Publisher’s note

All claims expressed in this article are solely those of the authors and do not necessarily represent those of their affiliated organizations, or those of the publisher, the editors and the reviewers. Any product that may be evaluated in this article, or claim that may be made by its manufacturer, is not guaranteed or endorsed by the publisher.
