# Supplementary material for: Cancer chemotherapy: insights into cellular and tumor microenvironmental mechanisms of action
Source: Front Oncol. 2022 Jul 29;12:960317. doi: 10.3389/fonc.2022.960317 (PMC9372369; doi:10.3389/fonc.2022.960317)
Supplement: Supplementary file 1 [file Table_1.docx]

Supplementary Material

# Supplementary tables

**Supplementary Table 1.** Studies examining the relationship between Ki67 expression before chemotherapy treatment with response and survival. RTX, radiotherapy. pCR pathological complete response. OS, overall survival. PFS, progression free survival, RFS, relapse free survival. 5-FU, 5-flurouracil

| **Cancer** | **Treatment** | **Response Rate** | **Survival** | **Ref** |
| --- | --- | --- | --- | --- |
| Neuroendocrine carcinoma (GI) | Platinum based combinations | Lower response rate when Ki67<55% (15%) compared to Ki67>55% (42%) | Ki67<55% improved OS | (1) |
| Neuroendocrine carcinoma (Pancreatic) | Platinum/etoposide,  Streptozocin/5-FU  Streptozocin/5-FU/ platinum | Trend towards lower response rate when Ki67<35% compared | NA | (2) |
|  | Streptozocin/5-FU | No correlation | Lower OS when Ki67>15% | (3) |
|  | Platinum based combinations | NA | Ki67>55% poorer survival | (4) |
|  | Streptozocin/5-FU or doxorubicin | No correlation | Ki67>10% poorer PFS and OS | (5) |
| Neuroendocrine Carcinoma (various) | Platinum based combinations | NA | No correlation. | (6) |
|  | Cisplatin/etoposide  Carboplatin/etoposide | NA | Ki67<55% improved OS and PFS | (7) |
|  | Platinum based combinations | No correlation | No correlation | (8) |
|  | Cisplatin/5-FU/streptozocin | No correlation | No correlation | (9) |
| Neuroendocrine Carcinoma (Extrapulmonary) | Platinum based combinations | No correlation | No correlation | (10) |
| SCLC | Platinum based combinations | No correlation | No correlation | (10) |
|  | Cisplatin/etoposide/RTX  Carboplatin/etoposide/RTX | Higher Ki67 (>79%) improved rate of pCR | No correlation | (11) |
|  | Platinum based combinations | NA |  | (12) |
| Non Small Cell Lung Cancer | Platinum based combinations | No correlation | NA | (13) |
|  | Cisplatin based chemotherapy | NA | No correlation | (14) |
|  | Docetaxel/gemcitabine/paclitaxel with or without cisplatin/carboplatin. | NA | No correlation | (15) |
|  | Platinum based combinations | No correlation | No correlation | (16) |
|  | Cisplatin based chemotherapy | NA | No correlation | (17) |
|  | Cisplatin based chemotherapy | No correlation | No correlation | (18) |
|  | Cisplatin based chemotherapy | No correlation | Positive Ki67 poorer PFS and OS | (19) |
| Pancreatic Cancer | 81% received chemotherapy | NA | No correlation. | (20) |
|  | Gemcitabine | NA | Higher Ki67 (>13%) poorer PFS and OS | (21) |
|  | Gemcitabine/cisplatin | No correlation | NA | (22) |
| Mesothelioma (MPeriM) | Cisplatin/doxorubicin | NA | Poor prognosis when Ki67 high (>25%) | (23) |
|  | Cisplatin/doxorubicin  Cisplatin/mitomycin C | NA | Improved OS with low Ki67 (<9%) | (24) |
|  | Cisplatin/doxorubicin  Cisplatin/mitomycin C | NA | Improved OS and PFS with low Ki67 (<10%) | (25) |
| Colon Cancer | 5-FU | NA | No correlation | (26) |
|  | 5-FU alone or in combinations | NA | No correlation | (27) |
|  | 5-FU based (neoadjuvant) | NA | Ki67>40% improved RFS and | (28) |
| Rectal Cancer | 5-FU Based (neoadjuvant) | NA | No correlation | (28) |
|  | CRT (did not specify regime) | No correlation | NA | (29) |
|  | Uracil/tegafur/RTX | No correlation | No correlation | (30) |
|  | Oxaliplatin/capecitabine/RTX | Higher Ki67 improved tumor response grade | No correlation | (31) |
|  | 5-FU/RTX | No correlation | No correlation | (32) |
|  | 5-FU/RTX | Low Ki67 associated with high tumor response | NA | (33) |
|  | 5-FU/RTX | No correlation | No correlation | (34) |
|  | 5-FU+RTX  5-FU/RTX/oxaliplatin | Higher Ki67 improved response rate | No correlation | (35) |
|  | 5-FU/camptothecin-11/RTX | No correlation | NA | (36) |
|  | 5-FU/RTX | No correlation | NA | (37) |
|  | 5-FU/RTX | No correlation | NA | (38) |
|  | 5-FU/RTX | Higher Ki67 improved response rate | NA | (38) |
| Head and Neck Cancer | Cisplatin/5-FU followed by Cisplatin/RTX | No correlation | No correlation | (39) |
|  | Platinum/5-FU/cetuximab | NA | No correlation | (40) |
|  | 5-FU/mitomycin-C/RTX | No correlation | No correlation | (41) |
|  | 5-FU/IFNα  5-FU/methotrexate  Carboplatin/4‑epaidriamycin/tenoposide | No correlation | No correlation | (42) |
|  | Doxetaxel/5-FU/cisplatin | No correlation | No correlation | (43) |
|  | Cisplatin/5-FU/RTX | Higher Ki67 in patients with a pCR | Higher Ki67 (>50%) improved survival | (44) |
|  | Nab‑paclitaxel/cisplatin/5‑FU/certuximab  Docetaxel/cisplatin/5-FU ±cetuximab | No correlation | No correlation | (45) |
|  | Oxaliplatin/S-1 | Higher Ki67 in patients that responded | NA | (46) |
|  | 5-FU/cisplatin/docetaxel/RTX  Cisplatin/docetaxel/RTX  Docetaxel/RTX | NA | No correlation | (47) |
|  | 5-FU/cisplatin/RTX | No correlation | Ki67+ had poorer OS. | (48) |
|  | 5-FU/cisplatin/RTX | No correlation | NA | (49) |

**Supplementary Table 2.** Clinical studies investigating the change in the TME (tumor biopsies) before and after chemotherapy treatment. Analysis of studies that monitor the expression/presence of markers or cell types before and after chemotherapy treatment and correlate the change in expression over the course of treatment with clinical outcome. AI, apoptotic index. DFS, disease free survival. NAC, neoadjuvant chemotherapy. OS, overall survival. pCR, pathological complete response. PD, progressive disease. PFS, progression free survival. PR, partial response. RFS, relapse free survival. SD, stable disease.

| **Study** | **Marker** | **Pre-treatment expression and correlation with response** | **Change in expression**  **(pre to post chemotherapy)** | **Correlation of change in expression with clinical response?** |
| --- | --- | --- | --- | --- |
| **Breast Cancer** | | | | |
| Eralp et.al (50) | GSTP1 | - | Decreased | Decrease correlated with response  Decrease associated with PFS, not OS |
|  | TOP2a | - | No change | No correlation |
|  | Survivin | - | No change | No correlation |
| Chuah et.al (51) | COX-2 | No correlation OS/PFS | Decreased | Decrease more prominent in responders  COX-2 negative pre- and post- treatment better PFS  COX-2 positive throughout poorer PFS |
|  | EGFR | No correlation | No change | No correlation |
|  | ErbB2 | Higher expression correlated with worse PFS | No change | No correlation |
|  | Ki67 | No correlation | No change | No correlation |
|  | p53 | No correlation | No change | No correlation |
| Burcombe et.al (52) | Ki67 | - | Decreased in 70% | Responders lower Ki67 and greater reduction from baseline |
|  | A) | - | Increased in 50% and same in 45% | Increased AI is a predictor pathological response, no correlation clinical response or PFS/OS |
| Hӧglander et.al (53) | Genomic aberrations (Instability) | Higher in good responders compared to intermediate or non responders | Decreased | Levels dropped in good responders while modest or no drop in non responders. |
| Alamgeer et.al (54) | ALDH1 | No correlation with OS | Increased in patients with no pCR | ALDH1+ tumor at baseline or midpoint had poorer response rate. If negative at baseline and midpoint better outcome.  ALDH1– tumor after treatment better OS |
| Nishimura et.al (55) | Ki67 | Higher Ki67 in pCR | Decreased from 44% to 16.5% after NAC | Patients who had pCR have lower Ki67 after chemotherapy when compared to PR, SD and PD. Patients with lower Ki67 after chemotherapy has better DFS |
| Chen et.al (56) | Ki67 | Higher in pCR but did not correlate to RFS or OS | Decreased after NAC from median 30% to 20% (p<0.001). Decrease predominantly in RF groups | Patients with Ki67 decrease >12.5% had better RFS. Ki67 expression change and histological grade significant in multi-variate analysis for RFS. |
| Von Minckwitz et.al (57) | Ki67 |  | Both decreased and increased dependent on response | Low post-treatment Ki67 has more favourable outcome regardless of pre-treatment levels. Post-treatment Ki67 more informative then pre-treatment or change |
| Bottini et.al (58) | Ki67 | No correlation | Decreased | Ki67 reduction greater in those with a clinical response then PR,SD. |
| Cabrera-Galeana et.al (59) | Ki67 | NA | 57% had decreased Ki67. | Patients with a decrease in Ki67 has longer DFS, 3 year DFS, OS. Difference in Ki67 significant determinant of survival using multivariate analysis. |
| Tokuda et.al (60) | Ki67 | No correlation in pre-treatment KI67 with development of metastasis |  | Mean Ki67 level higher after CTX higher in patients with metastasis. Lower Ki67 expression in the post-CTX sample compared to pre-CTX in patients with no metstasis. |
| Archer et.al (61) | Ki67 | Higher proliferation correlated with increased clinical response |  | No correlation |
|  | AI | No correlation | Increased | No correlation |
| Ellis et.al (62) | Ki67 | No correlation | Decreased or unchanged | No correlation |
|  | AI | No correlation | Decreased or unchanged | No correlation |
|  | BCL-2 | No correlation | Increased (negative->positive) | No correlation |
| Billgren et.al (63) | Ki67 |  |  | Decrease of proliferating fraction of cells >25% in patients with no recurrence. Higher RFS and OS. |
| Lee et.al (64) | Ki67 | No correlation | Median decreased | No correlation with clinical or pathological response. |
| Colleoni et.al (65) | Ki67 | Higher Ki67 in pCR | - | No correlation |
| Faneyte et.al (66) | BCL-2 | No correlation | No difference | No correlation |
|  | Ki67 | Higher level correlated with response. Not correlated with DFS or OS | Decreased | No correlation |
|  | P53 | Positively correlated with response. Not correlated with DFS or OS | No difference | No correlation |
| **Non Small Cell Lung Cancer** | | | | |
| Sheng et.al (67) | PD-L1 | - | Decreased on tumor cells | Decrease on tumor cells correlated with good response  Switching from PD-L1 positive to negative had better DFS. |
| **Mesothelioma** | | | | |
| Bitanihirwe et.al (68) | PTEN | No correlation | Decreased after chemotherapy | Reduction in expression from pre-post chemotherapy has shorter OS but not PFS |
|  | Phospho-S6 | Lower expression, increased PFS | Decreased after chemotherapy | No correlation |
|  | Phosphor-mTOR | No correlation | Decreased after chemotherapy | Increased expression pre- post chemotherapy shorter OS but not PFS |
|  | Cleaved Caspase 3 (AI) | No correlation | Increased after chemotherapy | No correlation |
|  | Ki67 | Lower expression, increased PFS | Decreased after chemotherapy | Lower expression after chemotherapy, increased PFS and OS. No correlation in reduction/changes of expression. |
| **Rectal Carcinoma** | | | | |
| Havelund et. al (69) | HIF-1a | No correlation | Decreased | No correlation |
|  | GLUT-1 | No correlation | Increased early, then returned to baseline | No correlation |
|  | Bcl-2 | No correlation | Decreased | No correlation |
|  | Ki-67 | No correlation | Decreased | No correlation |
| Debucquoy et. al (70) | COX-2 | No correlation | Increased after chemotherapy | No correlation |
|  | Ki-67 | No correlation | Decreased after chemotherapy | No correlation |

# Supplementary references

1. Sorbye H, Welin S, Langer SW, Vestermark LW, Holt N, Osterlund P, et al. Predictive and prognostic factors for treatment and survival in 305 patients with advanced gastrointestinal neuroendocrine carcinoma (WHO G3): the NORDIC NEC study. Ann Oncol. 2013 Jan;24(1):152–60.

2. Childs A, Kirkwood A, Edeline J, Luong TV, Watkins J, Lamarca A, et al. Ki-67 index and response to chemotherapy in patients with neuroendocrine tumours. Endocr Relat Cancer. 2016;23(7):563–70.

3. Dilz LM, Denecke T, Steffen IG, Prasad V, von Weikersthal LF, Pape UF, et al. Streptozocin/5-fluorouracil chemotherapy is associated with durable response in patients with advanced pancreatic neuroendocrine tumours. Eur J Cancer. 2015 Jul;51(10):1253–62.

4. Hijioka S, Hosoda W, Matsuo K, Ueno M, Furukawa M, Yoshitomi H, et al. Rb Loss and KRAS Mutation Are Predictors of the Response to Platinum-Based Chemotherapy in Pancreatic Neuroendocrine Neoplasm with Grade 3: A Japanese Multicenter Pancreatic NEN-G3 Study. Clin Cancer Res. 2017 Aug 15;23(16):4625–32.

5. Krug S, Boch M, Daniel H, Nimphius W, Müller D, Michl P, et al. Streptozocin-Based Chemotherapy in Patients with Advanced Neuroendocrine Neoplasms--Predictive and Prognostic Markers for Treatment Stratification. PLoS ONE. 2015;10(12):e0143822.

6. Spada F, Antonuzzo L, Marconcini R, Radice D, Antonuzzo A, Ricci S, et al. Oxaliplatin-Based Chemotherapy in Advanced Neuroendocrine Tumors: Clinical Outcomes and Preliminary Correlation with Biological Factors. Neuroendocrinology. 2016;103(6):806–14.

7. Bongiovanni A, Riva N, Ricci M, Liverani C, La Manna F, De Vita A, et al. First-line chemotherapy in patients with metastatic gastroenteropancreatic neuroendocrine carcinoma. Onco Targets Ther. 2015;8:3613–9.

8. Özaslan E, Karaca H, Koca S, Sevinç A, Hacioğlu B, Özkan M, et al. Comparison of survival with somatostatin analog and chemotherapy and prognostic factors for treatment in 165 advanced neuroendocrine tumor patients with Ki-67 20% or less. Anticancer Drugs. 2017;28(2):222–9.

9. Turner NC, Strauss SJ, Sarker D, Gillmore R, Kirkwood A, Hackshaw A, et al. Chemotherapy with 5-fluorouracil, cisplatin and streptozocin for neuroendocrine tumours. Br J Cancer. 2010 Mar 30;102(7):1106–12.

10. de M Rêgo JF, de Medeiros RSS, Braghiroli MI, Galvão B, Neto JEB, Munhoz RR, et al. Expression of ERCC1, Bcl-2, Lin28a, and Ki-67 as biomarkers of response to first-line platinum-based chemotherapy in patients with high-grade extrapulmonary neuroendocrine carcinomas or small cell lung cancer. Ecancermedicalscience [Internet]. 2017 Sep 11 [cited 2019 Nov 13];11. Available from: https://www.ncbi.nlm.nih.gov/pmc/articles/PMC5606295/

11. Ishibashi N, Maebayashi T, Aizawa T, Sakaguchi M, Nishimaki H, Masuda S. Correlation between the Ki-67 proliferation index and response to radiation therapy in small cell lung cancer. Radiat Oncol [Internet]. 2017 Jan 13 [cited 2019 Nov 13];12. Available from: https://www.ncbi.nlm.nih.gov/pmc/articles/PMC5237196/

12. Skov BG, Holm B, Erreboe A, Skov T, Mellemgaard A. ERCC1 and Ki67 in small cell lung carcinoma and other neuroendocrine tumors of the lung: distribution and impact on survival. J Thorac Oncol. 2010 Apr;5(4):453–9.

13. Mohamed S, Yasufuku K, Nakajima T, Hiroshima K, Kubo R, Iyoda A, et al. Analysis of cell cycle-related proteins in mediastinal lymph nodes of patients with N2-NSCLC obtained by EBUS-TBNA: relevance to chemotherapy response. Thorax. 2008 Jul;63(7):642–7.

14. Filipits M, Pirker R, Dunant A, Lantuejoul S, Schmid K, Huynh A, et al. Cell cycle regulators and outcome of adjuvant cisplatin-based chemotherapy in completely resected non-small-cell lung cancer: the International Adjuvant Lung Cancer Trial Biologic Program. J Clin Oncol. 2007 Jul 1;25(19):2735–40.

15. Yan S, Shun-Chang J, Li C, Jie L, Ya-Li L, Ling-Xiong W. Topoisomerase II alpha expression and the benefit of adjuvant chemotherapy for postoperative patients with non-small cell lung cancer. BMC Cancer. 2010 Nov 10;10(1):621.

16. Dingemans AMC, van Ark-Otte J, Span S, Scagliotti GV, van der Valk P, Postmus PE, et al. Topoisomerase IIα and other drug resistance markers in advanced non-small cell lung cancer. Lung Cancer. 2001 May 1;32(2):117–28.

17. Warth A, Cortis J, Soltermann A, Meister M, Budczies J, Stenzinger A, et al. Tumour cell proliferation (Ki-67) in non-small cell lung cancer: a critical reappraisal of its prognostic role. Br J Cancer. 2014 Sep 9;111(6):1222–9.

18. van de Vaart PJ, Belderbos J, de Jong D, Sneeuw KC, Majoor D, Bartelink H, et al. DNA-adduct levels as a predictor of outcome for NSCLC patients receiving daily cisplatin and radiotherapy. Int J Cancer. 2000 Mar 20;89(2):160–6.

19. Kaira K, Takahashi T, Murakami H, Shukuya T, Kenmotsu H, Ono A, et al. The role of βIII-tubulin in non-small cell lung cancer patients treated by taxane-based chemotherapy. Int J Clin Oncol. 2013 Jun;18(3):371–9.

20. Qin R, Smyrk TC, Reed NR, Schmidt RL, Schnelldorfer T, Chari ST, et al. Combining clinicopathological predictors and molecular biomarkers in the oncogenic K-RAS/Ki67/HIF-1α pathway to predict survival in resectable pancreatic cancer. Br J Cancer. 2015 Feb 3;112(3):514–22.

21. Striefler JK, Sinn M, Pelzer U, Jühling A, Wislocka L, Bahra M, et al. P53 overexpression and Ki67-index are associated with outcome in ductal pancreatic adenocarcinoma with adjuvant gemcitabine treatment. Pathology - Research and Practice. 2016 Aug 1;212(8):726–34.

22. Heinrich S, Schäfer M, Weber A, Hany TF, Bhure U, Pestalozzi BC, et al. Neoadjuvant chemotherapy generates a significant tumor response in resectable pancreatic cancer without increasing morbidity: results of a prospective phase II trial. Ann Surg. 2008 Dec;248(6):1014–22.

23. Pillai K, Pourgholami MH, Chua TC, Morris DL. Prognostic significance of Ki67 expression in malignant peritoneal mesothelioma. Am J Clin Oncol. 2015 Aug;38(4):388–94.

24. Kusamura S, Torres Mesa PA, Cabras A, Baratti D, Deraco M. The Role of Ki-67 and Pre-cytoreduction Parameters in Selecting Diffuse Malignant Peritoneal Mesothelioma (DMPM) Patients for Cytoreductive Surgery (CRS) and Hyperthermic Intraperitoneal Chemotherapy (HIPEC). Ann Surg Oncol. 2016 May;23(5):1468–73.

25. Baratti D, Kusamura S, Cabras AD, Bertulli R, Hutanu I, Deraco M. Diffuse malignant peritoneal mesothelioma: long-term survival with complete cytoreductive surgery followed by hyperthermic intraperitoneal chemotherapy (HIPEC). Eur J Cancer. 2013 Oct;49(15):3140–8.

26. Flanagan L, Meyer M, Fay J, Curry S, Bacon O, Duessmann H, et al. Low levels of Caspase-3 predict favourable response to 5FU-based chemotherapy in advanced colorectal cancer: Caspase-3 inhibition as a therapeutic approach. Cell Death Dis. 2016 Feb;7(2):e2087.

27. Allegra CJ, Parr AL, Wold LE, Mahoney MR, Sargent DJ, Johnston P, et al. Investigation of the prognostic and predictive value of thymidylate synthase, p53, and Ki-67 in patients with locally advanced colon cancer. J Clin Oncol. 2002 Apr 1;20(7):1735–43.

28. Fluge Ø, Gravdal K, Carlsen E, Vonen B, Kjellevold K, Refsum S, et al. Expression of EZH2 and Ki-67 in colorectal cancer and associations with treatment response and prognosis. Br J Cancer. 2009 Oct 20;101(8):1282–9.

29. Kundel Y, Nasser NJ, Rath-Wolfson L, Purim O, Yanichkin N, Brenner R, et al. Molecular Predictors of Response to Neoadjuvant Chemoradiation for Rectal Cancer. Am J Clin Oncol. 2018;41(6):613–8.

30. Suzuki T, Sadahiro S, Tanaka A, Okada K, Saito G, Kamijo A, et al. Predictive markers of chemoradiotherapy for rectal cancer: comparison of biopsy specimens taken before and about 1 week after the start of chemoradiotherapy. Int J Clin Oncol. 2015 Dec;20(6):1130–9.

31. Carlomagno C, Pepe S, D’Armiento FP, D’Armiento M, Cannella L, De Stefano A, et al. Predictive factors of complete response to neoadjuvant chemoradiotherapy in patients with rectal cancer. Oncology. 2010;78(5–6):369–75.

32. Terzi C, Canda AE, Sagol O, Atila K, Sonmez D, Fuzun M, et al. Survivin, p53, and Ki-67 as predictors of histopathologic response in locally advanced rectal cancer treated with preoperative chemoradiotherapy. Int J Colorectal Dis. 2008 Jan;23(1):37–45.

33. Jakob C, Liersch T, Meyer W, Becker H, Baretton GB, Aust DE. Predictive value of Ki67 and p53 in locally advanced rectal cancer: correlation with thymidylate synthase and histopathological tumor regression after neoadjuvant 5-FU-based chemoradiotherapy. World J Gastroenterol. 2008 Feb 21;14(7):1060–6.

34. Reerink O, Karrenbeld A, Plukker JTM, Verschueren RCJ, Szabó BG, Sluiter WJ, et al. Molecular prognostic factors in locally irresectable rectal cancer treated preoperatively by chemo-radiotherapy. Anticancer Res. 2004 Apr;24(2C):1217–21.

35. Rödel C, Grabenbauer GG, Papadopoulos T, Bigalke M, Günther K, Schick C, et al. Apoptosis as a cellular predictor for histopathologic response to neoadjuvant radiochemotherapy in patients with rectal cancer. Int J Radiat Oncol Biol Phys. 2002 Feb 1;52(2):294–303.

36. Charara M, Edmonston TB, Burkholder S, Walters R, Anne P, Mitchell E, et al. Microsatellite status and cell cycle associated markers in rectal cancer patients undergoing a combined regimen of 5-FU and CPT-11 chemotherapy and radiotherapy. Anticancer Res. 2004 Oct;24(5B):3161–7.

37. Smith FM, Reynolds JV, Kay EW, Crotty P, Murphy JO, Hollywood D, et al. COX-2 overexpression in pretreatment biopsies predicts response of rectal cancers to neoadjuvant radiochemotherapy. Int J Radiat Oncol Biol Phys. 2006 Feb 1;64(2):466–72.

38. Chang HJ, Jung KH, Kim DY, Jeong SY, Choi HS, Kim YH, et al. Bax, a predictive marker for therapeutic response to preoperative chemoradiotherapy in patients with rectal carcinoma. Hum Pathol. 2005 Apr;36(4):364–71.

39. Rodriguez-Pinilla M, Rodriguez-Peralto JL, Hitt R, Sanchez JJ, Ballestin C, Diez A, et al. Cyclin A as a predictive factor for chemotherapy response in advanced head and neck cancer. Clin Cancer Res. 2004 Dec 15;10(24):8486–92.

40. Vermorken JB, Peyrade F, Krauss J, Mesía R, Remenar E, Gauler TC, et al. Cisplatin, 5-fluorouracil, and cetuximab (PFE) with or without cilengitide in recurrent/metastatic squamous cell carcinoma of the head and neck: results of the randomized phase I/II ADVANTAGE trial (phase II part). Ann Oncol. 2014 Mar;25(3):682–8.

41. Perisanidis C, Perisanidis B, Wrba F, Brandstetter A, El Gazzar S, Papadogeorgakis N, et al. Evaluation of immunohistochemical expression of p53, p21, p27, cyclin D1, and Ki67 in oral and oropharyngeal squamous cell carcinoma. J Oral Pathol Med. 2012 Jan;41(1):40–6.

42. Heeren P a. M, Kloppenberg FWH, Hollema H, Mulder NH, Nap RE, Plukker JTM. Predictive Effect of p53 and p21 Alteration on Chemotherapy Response and Survival in Locally Advanced Adenocarcinoma of the Esophagus. Anticancer Res. 2004 Jul 1;24(4):2579–84.

43. Yamamoto Y, Yamai H, Seike J, Yoshida T, Takechi H, Furukita Y, et al. Prognosis of esophageal squamous cell carcinoma in patients positive for human epidermal growth factor receptor family can be improved by initial chemotherapy with docetaxel, fluorouracil, and cisplatin. Ann Surg Oncol. 2012 Mar;19(3):757–65.

44. Takeuchi H, Ozawa S, Ando N, Kitagawa Y, Ueda M, Kitajima M. Cell-cycle regulators and the Ki-67 labeling index can predict the response to chemoradiotherapy and the survival of patients with locally advanced squamous cell carcinoma of the esophagus. Ann Surg Oncol. 2003 Aug;10(7):792–800.

45. Chatzkel J, Lewis JS, Ley JC, Wildes TM, Thorstad W, Gay H, et al. Correlation of Ki-67 Proliferative Antigen Expression and Tumor Response to Induction Chemotherapy Containing Cell Cycle-Specific Agents in Head and Neck Squamous Cell Carcinoma. Head Neck Pathol. 2017 Sep;11(3):338–45.

46. Li S, Li B, Wang J, Zhang D, Liu Z, Zhang Z, et al. Identification of Sensitivity Predictors of Neoadjuvant Chemotherapy for the Treatment of Adenocarcinoma of Gastroesophageal Junction. Oncol Res. 2017 Jan 2;25(1):93–7.

47. Maebayashi T, Ishibashi N, Aizawa T, Sakaguchi M, Saito T, Kawamori J, et al. Roles of Ki-67 and p16 as biomarkers for unknown primary head and neck squamous cell carcinoma. Eur Arch Otorhinolaryngol. 2019 Apr;276(4):1221–9.

48. Lavertu P, Adelstein DJ, Myles J, Secic M. P53 and Ki-67 as outcome predictors for advanced squamous cell cancers of the head and neck treated with chemoradiotherapy. Laryngoscope. 2001 Nov;111(11 Pt 1):1878–92.

49. Sohda M, Ishikawa H, Masuda N, Kato H, Miyazaki T, Nakajima M, et al. Pretreatment evaluation of combined HIF-1alpha, p53 and p21 expression is a useful and sensitive indicator of response to radiation and chemotherapy in esophageal cancer. Int J Cancer. 2004 Jul 20;110(6):838–44.

50. Eralp Y, Keskin S, Akışık E, Akışık E, İğci A, Müslümanoğlu M, et al. Predictive role of midtreatment changes in survivin, GSTP1, and topoisomerase 2α expressions for pathologic complete response to neoadjuvant chemotherapy in patients with locally advanced breast cancer. Am J Clin Oncol. 2013 Jun;36(3):215–23.

51. Chuah BYS, Putti T, Salto-Tellez M, Charlton A, Iau P, Buhari SA, et al. Serial changes in the expression of breast cancer-related proteins in response to neoadjuvant chemotherapy. Ann Oncol. 2011 Aug;22(8):1748–54.

52. Burcombe R, Wilson GD, Dowsett M, Khan I, Richman PI, Daley F, et al. Evaluation of Ki-67 proliferation and apoptotic index before, during and after neoadjuvant chemotherapy for primary breast cancer. Breast Cancer Res. 2006;8(3):R31.

53. Höglander EK, Nord S, Wedge DC, Lingjærde OC, Silwal-Pandit L, Gythfeldt H vdL, et al. Time series analysis of neoadjuvant chemotherapy and bevacizumab-treated breast carcinomas reveals a systemic shift in genomic aberrations. Genome Med. 2018 29;10(1):92.

54. Alamgeer M, Ganju V, Kumar B, Fox J, Hart S, White M, et al. Changes in aldehyde dehydrogenase-1 expression during neoadjuvant chemotherapy predict outcome in locally advanced breast cancer. Breast Cancer Res. 2014;16(2):R44.

55. Nishimura R, Osako T, Okumura Y, Hayashi M, Arima N. Clinical significance of Ki-67 in neoadjuvant chemotherapy for primary breast cancer as a predictor for chemosensitivity and for prognosis. Breast Cancer. 2010 Oct;17(4):269–75.

56. Chen C, Zhang Y, Huang Z, Wu J, Huang W, Zhang G. Decrease in the Ki67 index during neoadjuvant chemotherapy predicts favorable relapse-free survival in patients with locally advanced breast cancer. Cancer Biol Med. 2019 Aug;16(3):575–86.

57. von Minckwitz G, Schmitt WD, Loibl S, Muller BM, Blohmer JU, Sinn BV, et al. Ki67 Measured after Neoadjuvant Chemotherapy for Primary Breast Cancer. Clinical Cancer Research. 2013 Aug 15;19(16):4521–31.

58. Bottini A, Berruti A, Bersiga A, Brizzi MP, Bruzzi P, Aguggini S, et al. Relationship between tumour shrinkage and reduction in Ki67 expression after primary chemotherapy in human breast cancer. Br J Cancer. 2001 Oct;85(8):1106–12.

59. Cabrera‐Galeana P, Muñoz‐Montaño W, Lara‐Medina F, Alvarado‐Miranda A, Pérez‐Sánchez V, Villarreal‐Garza C, et al. Ki67 Changes Identify Worse Outcomes in Residual Breast Cancer Tumors After Neoadjuvant Chemotherapy. Oncologist. 2018 Jun;23(6):670–8.

60. Tokuda E, Horimoto Y, Arakawa A, Himuro T, Senuma K, Nakai K, et al. Differences in Ki67 expressions between pre- and post-neoadjuvant chemotherapy specimens might predict early recurrence of breast cancer. Hum Pathol. 2017;63:40–5.

61. Archer CD, Parton M, Smith IE, Ellis PA, Salter J, Ashley S, et al. Early changes in apoptosis and proliferation following primary chemotherapy for breast cancer. Br J Cancer. 2003 Sep 15;89(6):1035–41.

62. Ellis PA, Smith IE, Detre S, Burton SA, Salter J, A’Hern R, et al. Reduced apoptosis and proliferation and increased Bcl-2 in residual breast cancer following preoperative chemotherapy. Breast Cancer Res Treat. 1998 Mar;48(2):107–16.

63. Ann-Marie Billgren LER Edneia Tani, Nils Wilking, Tommy Fornander, Lambert Skoog, Ann Marie. Proliferating Fraction During Neoadjuvant Chemotherapy of Primary Breast Cancer in Relation to Objective Local Response and Relapse-free Survival. Acta Oncologica. 1999 Jan 1;38(5):597–601.

64. Lee J, Im YH, Lee SH, Cho EY, Choi YL, Ko YH, et al. Evaluation of ER and Ki-67 proliferation index as prognostic factors for survival following neoadjuvant chemotherapy with doxorubicin/docetaxel for locally advanced breast cancer. Cancer Chemother Pharmacol. 2008 Apr;61(4):569–77.

65. Colleoni M, Viale G, Zahrieh D, Pruneri G, Gentilini O, Veronesi P, et al. Chemotherapy is more effective in patients with breast cancer not expressing steroid hormone receptors: a study of preoperative treatment. Clin Cancer Res. 2004 Oct 1;10(19):6622–8.

66. Faneyte IF, Schrama JG, Peterse JL, Remijnse PL, Rodenhuis S, van de Vijver MJ. Breast cancer response to neoadjuvant chemotherapy: predictive markers and relation with outcome. Br J Cancer. 2003 Feb 10;88(3):406–12.

67. Sheng J, Fang W, Yu J, Chen N, Zhan J, Ma Y, et al. Expression of programmed death ligand-1 on tumor cells varies pre and post chemotherapy in non-small cell lung cancer. Sci Rep. 2016 Jan 29;6:20090.

68. Bitanihirwe BKY, Meerang M, Friess M, Soltermann A, Frischknecht L, Thies S, et al. PI3K/mTOR Signaling in Mesothelioma Patients Treated with Induction Chemotherapy Followed by Extrapleural Pneumonectomy. Journal of Thoracic Oncology. 2014 Feb 1;9(2):239–47.

69. Havelund BM, Sørensen FB, Pløen J, Lindebjerg J, Spindler KLG, Jakobsen A. Immunohistological expression of HIF-1α, GLUT-1, Bcl-2 and Ki-67 in consecutive biopsies during chemoradiotherapy in patients with rectal cancer. APMIS. 2013 Feb;121(2):127–38.

70. Debucquoy A, Goethals L, Geboes K, Roels S, Mc Bride WH, Haustermans K. Molecular responses of rectal cancer to preoperative chemoradiation. Radiother Oncol. 2006 Aug;80(2):172–7.
